# H19X-encoded microRNAs induced by IL-4 in adipocyte precursors regulate proliferation to facilitate differentiation

**DOI:** 10.1186/s13062-023-00388-4

**Published:** 2023-06-15

**Authors:** Choijamts Munkhzul, Ji-Min Lee, Boseon Kim, Thi Thanh My Nguyen, Rehna Paula Ginting, Dahee Jeong, Young-Kook Kim, Min-Woo Lee, Mihye Lee

**Affiliations:** 1 Soonchunhyang Institute of Medi-bio Science, Soonchunhyang University, Cheonan, 31151 Korea; 2 Department of Integrated Biomedical Science, Soonchunhyang University, Cheonan, 31151 Korea; 3 Department of Biochemistry, Chonnam National University Medical School, Hwasun, 58128 Korea

**Keywords:** Adipocyte precursor, Interleukin-4, microRNA, Beige adipocyte, Proliferation, Wnt signaling

## Abstract

Adipose tissue, an organ critical for systemic energy homeostasis, is influenced by type 2 immunity in its development and function. The type 2 cytokine interleukin (IL)-4 induces the proliferation of bipotential adipocyte precursors (APs) in white fat tissue and primes these cells for differentiation into beige adipocytes, which are specialized for thermogenesis. However, the underlying mechanisms have not yet been comprehensively examined. Here, we identified six microRNA (miRNA) genes upregulated upon IL-4 stimulation in APs, miR-322, miR-503, miR-351, miR-542, miR-450a, and miR-450b; these are encoded in the H19X locus of the genome. Their expression is positively regulated by the transcription factor *Klf4*, whose expression also increases upon IL-4 stimulation. These miRNAs shared a large set of target genes, of which 381 genes were downregulated in mRNA expression upon IL-4 stimulation and enriched in Wnt signaling pathways. Two genes with downregulated expression, *Ccnd1* and *Fzd6*, were repressed by H19X-encoded miRNAs. Additionally, the Wnt signaling activator LiCl downregulated the expression of this group of miRNAs in APs, indicating that Wnt signaling–related genes and these miRNAs form a double-negative feedback regulatory loop. This miRNA/Wnt feedback regulation modulated the elevated proliferation of APs induced by IL-4 stimulation and contributed to priming them for beige adipocyte differentiation. Moreover, the aberrant expression of these miRNAs attenuates the differentiation of APs into beige adipocytes. Collectively, our results suggest that H19X-encoded miRNAs facilitate the transition of APs from proliferation to differentiation in the IL-4-mediated regulation.

**Supplementary Information:**

The online version contains supplementary material available at 10.1186/s13062-023-00388-4.

## Introduction

Adipose tissue functions as a central organ for metabolic communication and control, thermoregulation, buffering of mechanical loads, and secretion of hormones [1, 2]. Distinct types of adipose tissue are generated from unique progenitor cells and differ in their roles [3–5]. White adipose tissue (WAT) consists of classical adipocytes with a single lipid droplet that stores energy as triacylglycerol [6–8]. WAT expands through hyperplasia and hypertrophy in response to excess energy uptake, which has profound effects on the size and number of adipocytes [9–11]. Brown adipose tissue is composed of multilocular cells with large amounts of mitochondria and elevated uncoupling protein 1 (UCP1) expression [12, 13]. It dissipates chemical energy as heat by UCP1-mediated uncoupling of respiration from ATP synthesis, termed non-shivering thermogenesis [14, 15]. The development and function of these different types of adipose tissue are critical for metabolic homeostasis, and their dysregulation underlies negative metabolic and health consequences [16]. Beige fat is another population of adipocytes with thermogenic capacity found in the subcutaneous WAT (scWAT) depot [17, 18]. Adrenergic stimulation promotes scWAT to gain brown fat-like features, such as elevated UCP1 levels and an increased number of mitochondria, upon external cues such as cold exposure [19, 20]. Thermogenic adipose tissue plays a protective role in metabolism through energy consumption, which has attracted interest in the browning of adipose tissue as a therapeutic strategy [21, 22]. Type 2 immunity is known to mediate metabolic adaptation in response to environmental cold temperatures [23, 24]. In particular, the type 2 cytokines IL-4 and IL-13 promote an increase in adipocyte precursors (APs) and their commitment to the beige adipocyte lineage, leading to the growth of beige fat [25]. However, the molecular mechanisms by which type 2 cytokines regulate APs during cold acclimatization are unclear.

MicroRNAs (miRNAs) are small non-coding RNAs (~ 22 nucleotides) that regulate gene expression at the post-transcriptional level [26, 27]. Mature miRNA is generated from the long primary transcript (pri-miRNA) through sequential cleavage by two RNase III enzymes, Drosha and Dicer [28–30]. The cleavage product, a small RNA duplex, is loaded onto Argonaute (Ago) protein, which preferentially retains one strand of the duplex [31, 32]. Mature miRNA binds the target mRNA through base-pairing and induces translational repression and degradation [33]. miRNAs are estimated to target more than 60% of the human protein-coding genes, indicating their regulatory involvement in diverse physiological and pathological contexts [34]. The binding sites of miRNA are predominantly located in the 3′ untranslated region (UTR) of target mRNAs and are recognized by the seed region that spans 2–7 nt at the 5′ end of a given miRNA [35, 36]. This binding, through partial complementarity, allows an individual miRNA to regulate hundreds of different mRNAs and consequently leads to the downregulation of large target cohorts. Although miRNAs modulate the expression levels of specific target genes, they exert systemic effects on the regulatory network, which is essential for a variety of cellular processes [37]. Additionally, miRNAs can provide robustness by accelerating changes in gene expression, particularly through the formation of feedback loops with their target genes [38, 39]. miRNAs also establish adaptability by balancing the dynamic expression ranges [40].miRNAs are critical regulators of adipose tissue development and function. The fat-specific knockout of Dicer and DGCR8, both of which are essential for the production of mature miRNAs, results in dramatic defects in adipose tissue formation and maintenance [41–43]. In addition, altered miRNA expression has been reported in different types of adipose tissues, during adipocyte differentiation, and under pathological conditions [44, 44, 45]. Multiple studies have shown that miRNAs constitute a regulatory network of gene expression that governs adipogenesis, including cell fate determination and adipocyte differentiation [46–50]. Accordingly, defects in miRNA-mediated regulation are involved in adipose tissue dysfunction and metabolic complications [51–53].

In this study, we investigated miRNA-mediated gene expression control in IL-4-induced priming of APs. Interleukin (IL)-4 stimulation leads to AP expansion, which then activates miRNA-mediated regulation of Wnt pathway genes. H19X-encoded miRNAs reinforce the suppression of the Wnt pathway through a double-negative feedback loop, which lowers the proliferation of APs and contributes to beige adipocyte commitment.

## Results

### Differential gene expression following IL-4 stimulation in APs

**Fig. 1:**
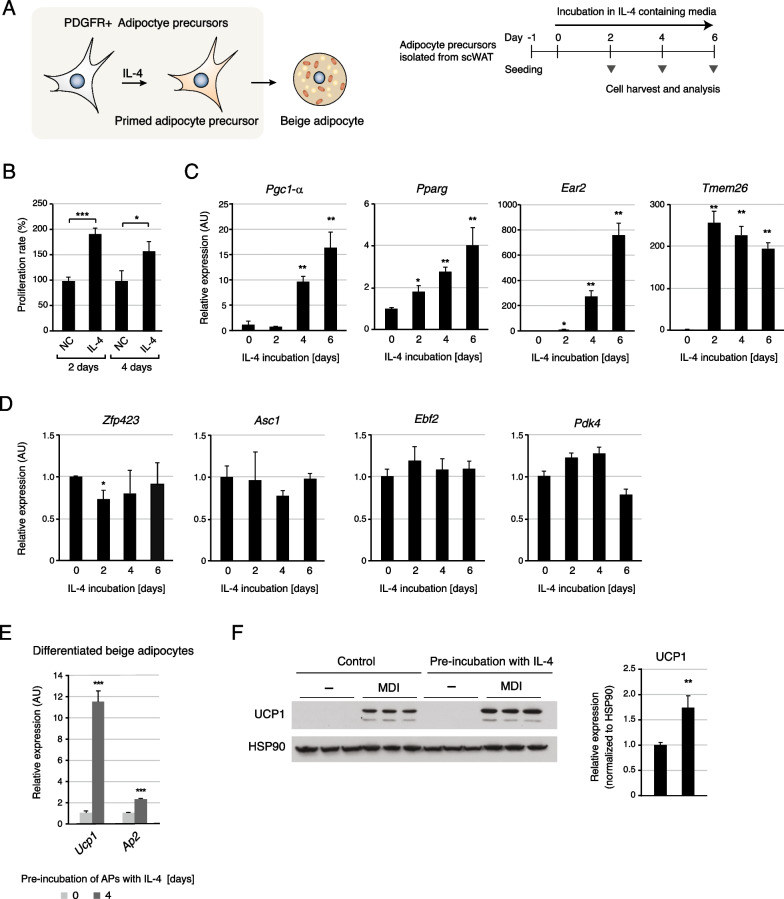
IL-4 promotes cellular expansion and beige commitment of adipocyte precursors (APs). **A** Schematic of IL-4 induced priming of APs. APs isolated from scWAT were cultured in vitro. IL-4 was added into the growth medium one day after cell seeding and cells were harvested for analysis on the indicated days **B** The cell proliferation rate of APs cultured in IL-4 containing media (n = 3). **C** The beige adipogenic markers (*Pgc1-α*, *Pparg*, *Ear2*, and *Tmem26*) mRNA levels were examined by qPCR in APs (n = 3). Longer incubation of APs with IL-4 induces higher expression levels of markers. **D** mRNA expression levels of the white adipogenic markers (*Zfp423* and *Asc1*) and the brown adipogenic markers (*Ebf2* and *Pdk4*) were analyzed by qPCR in APs (n = 3). **E** The mRNA levels of *Ucp1*, *Ap2*, and *Pgc1-α* genes were measured by quantitative PCR (qPCR) in differentiated adipocytes (n = 3). When APs were pre-incubated with IL-4 for four days before the induction of differentiation, the expressions of beige and adipogenic markers in differentiated adipocytes increased to a significantly greater extent than those in control cells. **F** The protein levels of UCP1 were examined by western blotting in differentiated adipocytes. Pre-incubation of APs with IL-4 leads to higher levels of UCP1 expression in differentiated adipocytes. Biological triplicates for each condition were examined. The quantified signal intensities in the western blotting are presented as a bar graph (n = 3). MDI refers to the induction of differentiation by an adipogenic cocktail. Data are presented as mean ± SEM. *** *p* < 0.005, **p < 0.01, *p < 0.05

The type 2 cytokine IL-4 has been identified as one of the key factors that regulate the number and fate of bipotential APs in white fat tissue [25]. However, the underlying gene expression changes have not been thoroughly examined, restricting further understanding of IL-4 signaling-mediated cellular processes in APs. We performed global analysis of gene expression altered by IL-4 stimulation in APs (Fig. 1A). We used APs isolated from the scWAT of C57BL/6 J mice and confirmed that IL-4 stimulation promoted cell proliferation and primed the precursors for beige adipocyte differentiation, consistent with previous in vivo results (Fig. 1A–C) [25, 54, 55]. The expression levels of beige adipogenic markers *Pgc1-α*, *Pparg*, *Ear2*, and *Tmem26* were markedly increased when APs were incubated with IL-4 (Fig. 1C). However, the expression levels of white or brown specific adipogenic markers did not significantly increase upon IL4 stimulation (Fig. 1D). To verify the effects of IL-4 on differentiation toward beige adipocytes, the differentiation was induced in IL-4-pretreated APs with an adipogenic cocktail. Notably, mature beige adipocytes were examined using *Ap2* and *Ucp1* expression levels, while the commitment of precursors to the beige lineage was analyzed using *Pgc1-α*, *Pparg*, *Ear2* and *Tmem26* expression levels, due to their different sensitivities in each context. The expression of adipogenic marker *Ap2* and beige adipocyte marker *Ucp1* was induced upon differentiation, with significantly higher levels in IL-4 pretreated cells than in control cells (Fig. 1E). Consistent with the upregulation at the mRNA level, UCP1 protein expression was also upregulated by IL-4 pretreatment (Fig. 1F). These results indicate that IL-4 leads to adaptive differentiation of APs to beige adipocytes by inducing the expressions of genes related to both cell proliferation and adipogenesis.

**Fig. 2:**
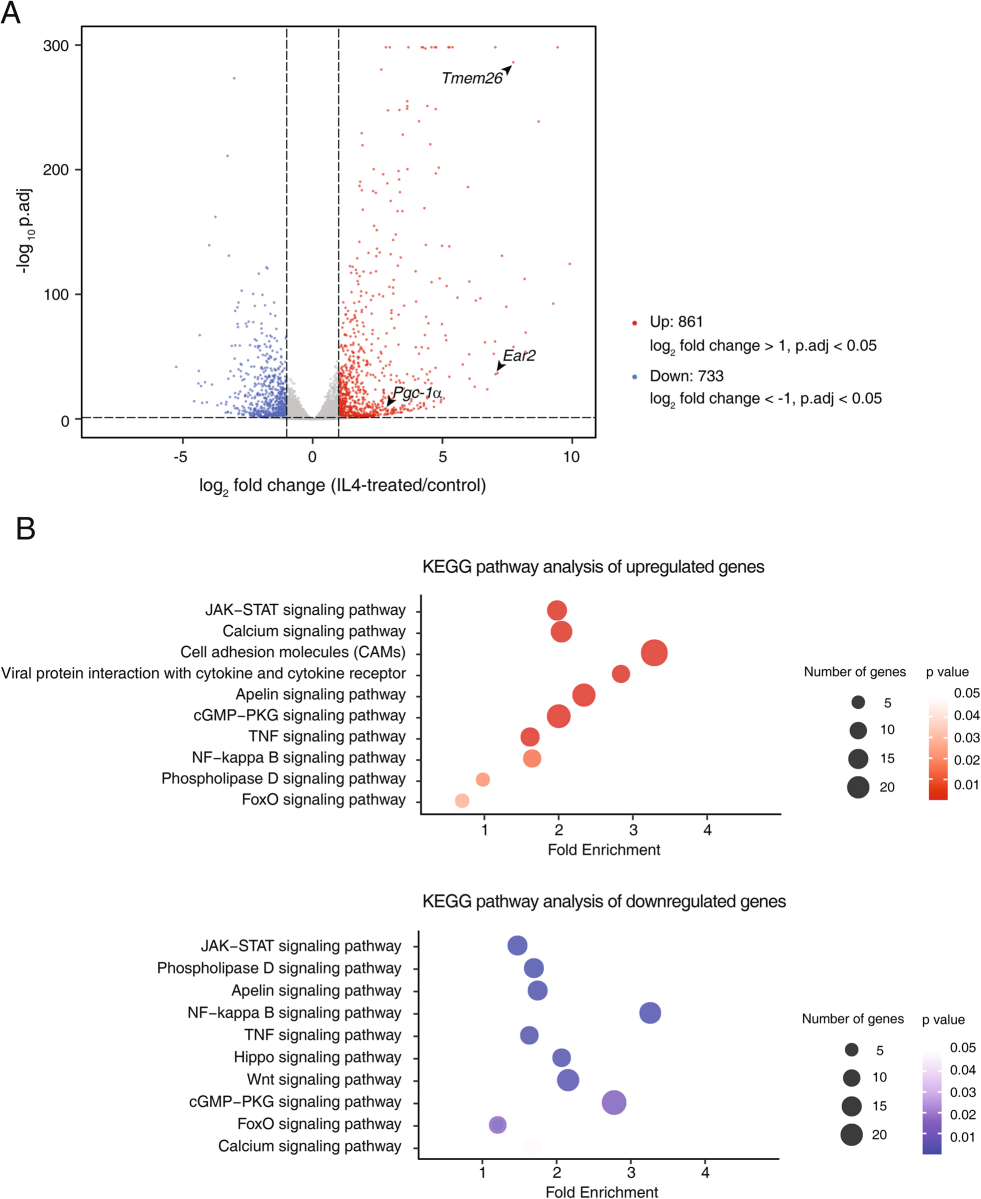
Differentially expressed genes in IL-4–treated APs. **A** Transcriptomic analysis of control and IL-4–treated APs (four-day sample). The mRNA-seq results are presented as a volcano plot (Total 18,345 genes). The genes of log2 fold change > 1 (red) or < -1 (blue) with p.adj < 0.05 are highlighted. *Pgc1-α*, *Ear2*, and *Tmem26* are labeled as the beige adipogenic markers. **B** KEGG pathway analysis of upregulated (log2 fold change > 1, p.adj < 0.05, upper panel, red) and downregulated (log2 fold change < -1, p.adj < 0.05, lower panel, blue) genes. The number of genes that belongs to each pathway is indicated by circle size and significance (p-value) is presented by color

High-throughput RNA sequencing revealed that APs incubated with IL-4 for four days showed prominent commitment to beige adipocyte differentiation (Fig. 2A, Additional file 1: Fig. S1, and Additional file 2: Table S1). At the transcriptomic level, the expressions of 861 genes were upregulated, and those of 733 genes were downregulated upon IL-4 stimulation (log2 fold change > 1 or < -1, p.adj < 0.05); these genes are commonly enriched in several signaling pathways, including JAK-STAT, NF-kappa B, phospholipase D, and calcium signaling. However, downregulated genes were specifically enriched in the Hippo and Wnt signaling pathways, which are critical for the regulation of cell proliferation (Fig. 2B). Through gene expression analysis at the transcriptomic level, a large set of genes were shown to be differentially expressed, suggesting that diverse signaling pathways are involved in the response to IL-4 stimulation.

### A group of microRNAs encoded in the H19X locus of the genome is upregulated immediately upon IL-4 stimulation

**Fig. 3:**
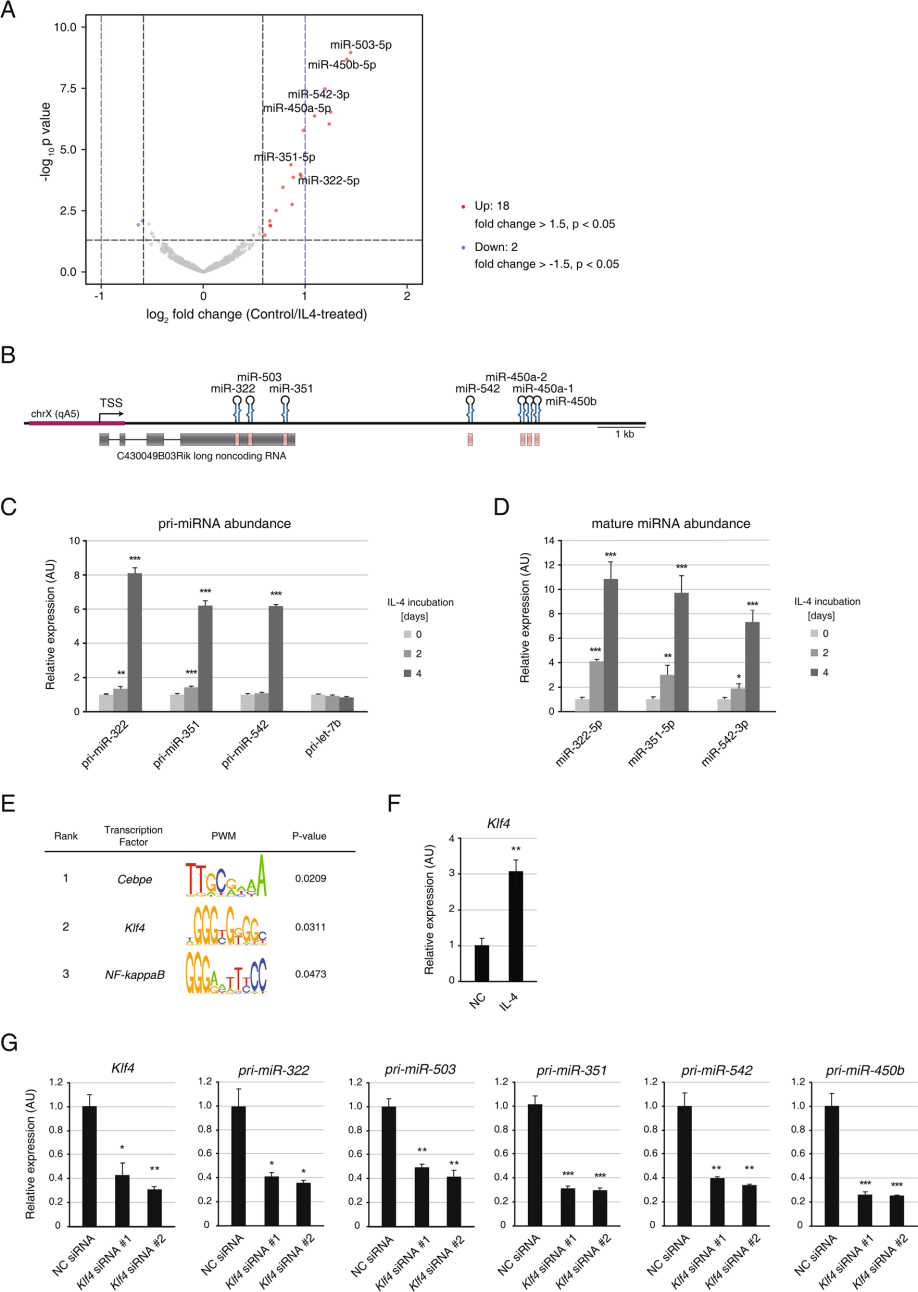
Differentially expressed miRNAs in IL-4–treated Aps. **A** The analysis of differentially expressed miRNAs using the small RNA sequencing data of control and IL-4–treated APs (two-day sample). Differentially expressed miRNAs in IL-4–treated APs are marked on the volcano plot as follows, fold change > 1.5 (red) or < -1.5 (blue), *p* < 0.05. The vertical dashed lines indicate the fold change of 1.5 (black) and 2.0 (blue). **B** The genomic structure of the H19X-encoded locus. miRNA genes are indicated by pink bars. The genomic region used for transcription factor enrichment analysis is marked with a red line. **C** The primary transcripts of *miR-322*, *miR-351*, and *miR-542* were examined by qPCR for the expression levels in APs incubated with IL-4 for 0, 2, and 4 days (n = 3). The *let-7b* primary transcript was used as a control. **D** The abundance of mature miRNAs, miR-322-5p, miR-351-5p, and miR-542-3p were examined by qPCR in APs incubated with IL-4 for 0, 2, and 4 days (n = 3). **E** Transcription factor enrichment analysis for the promoter region of H19X-encoded miRNAs. Top-ranked transcription factors are presented in the table. **F** *Klf4* mRNA levels were measured by qPCR in APs incubated with IL-4 for four days (n = 3). **G** *Klf4* mRNA and primary transcripts of *miR-322*, *miR-503, miR-351*, *miR-542*, and *miR-450b* were analyzed by qPCR for the expression levels after APs were treated with *Klf4* siRNA (n = 3). Data are presented as mean ± SEM. ***p < 0.005, **p < 0.01, *p < 0.05

miRNA is one of the important factors that constitute the regulatory network governing gene expression. Therefore, to reveal another layer of gene expression regulation, we analyzed the abundance of miRNAs on a global scale. For small RNA sequencing, we harvested APs incubated with IL-4 for two days, which allowed the detection of early response miRNAs. Six miRNAs were upregulated by > twofold upon IL-4 stimulation, and no miRNAs were downregulated by > twofold (Fig. 3A, Additional file 1: Fig. S2, and Additional file 3: Table S2). Eighteen upregulated and two downregulated miRNAs were found when a 1.5-fold change cut-off was applied (Fig. 3A and Additional file 3: Table S2). Of the 18 upregulated miRNAs, eleven were encoded by *miR-322*, *miR-503*, *miR-351*, *miR-542*, *miR-450a*, and *miR-450b* genes in the H19X locus of the genome (Fig. 3B and Table 1). To measure the levels of primary transcripts, the regions adjacent to the *miR-322, miR-351*, and *miR-542* hairpin structures were analyzed via quantitative RT-PCR. The levels of primary transcripts started to increase after two days of incubation with IL-4, albeit slightly, and increased by more than six-fold after four days (Fig. 3C). Mature miRNA levels increased by more than six-fold after four days of incubation with IL-4 (Fig. 3D). Together, these results indicate immediate transcriptional activation of H19X-encoded miRNAs upon IL-4 stimulation.

**Table 1:** Differentially expressed microRNAs in IL-4–stimulated adipocyte precursors

miRNA	Fold change	p-value	Genomic location	Major strand in miRBase
mmu-miR-503-5p	2.724024	1.10E-09	chrX: 53,053,984–53,054,054 [-]	*
mmu-miR-503-3p	1.845585	0.000137	chrX: 53,053,984–53,054,054 [-]	
mmu-miR-450b-5p	2.655063	2.06E-09	chrX: 53,047,997–53,048,078 [-]	*
mmu-miR-450b-3p	1.642237	0.003114	chrX: 53,047,997–53,048,078 [-]	
mmu-miR-351-5p	1.814575	4.21E-05	chrX: 53,053,255–53,053,353 [-]	*
mmu-miR-351-3p	2.379414	2.93E-07	chrX: 53,053,255–53,053,353 [-]	
mmu-miR-322-5p	1.944725	0.000121	chrX: 53,054,255–53,054,349 [-]	*
mmu-miR-322-3p	1.976286	1.65E-06	chrX: 53,054,255–53,054,349 [-]	
mmu-miR-542-5p	1.37794	0.050677	chrX: 53,049,403–53,049,487 [-]	
mmu-miR-542-3p	2.288134	3.33E-08	chrX: 53,049,403–53,049,487 [-]	*
mmu-miR-450a-5p	2.132565	4.26E-07	chrX: 53,048,154–53,048,244 [-]	*
mmu-miR-450a-1-3p	1.830931	0.001734	chrX: 53,048,154–53,048,244 [-]	
mmu-miR-450a-2-3p	1.219066	0.269456	chrX: 53,048,299–53,048,367 [-]	

Next, we searched for candidate transcription factors responsible for the upregulation of the H19X-encoded miRNAs. The transcription start site (TSS) for the *miR-322*, *miR-503*, *miR-351*, *miR-542*, *miR-450a*, and *miR-450b* genes was predicted by the microTSS machine learning algorithm developed by Georgakilas et al. [56], indicating chrX:53,057,195 in the mouse mm10 genome. We performed transcription factor enrichment analyses for the 2 kb surrounding the TSS sequences and found the transcription factors, *Cebpe*, *Klf4*, and *NF-kappaB* to be the most enriched (Fig. 3E). In particular, *Klf4* was significantly upregulated by IL-4 stimulation of the APs (Fig. 3F and Additional file 2: Table S1). The knockdown of *Klf4* resulted in the downregulation of the primary transcripts of H19X-encoded miRNAs including *miR-322, miR-503, miR-351, miR-542* and *miR-450b* (Fig. 3G). These results suggest that KLF4 transcriptionally activates the expression of H19X-encoded miRNAs in APs upon IL-4 stimulation.

### IL-4-upregulated miRNAs target Wnt signaling pathway genes

**Fig. 4:**
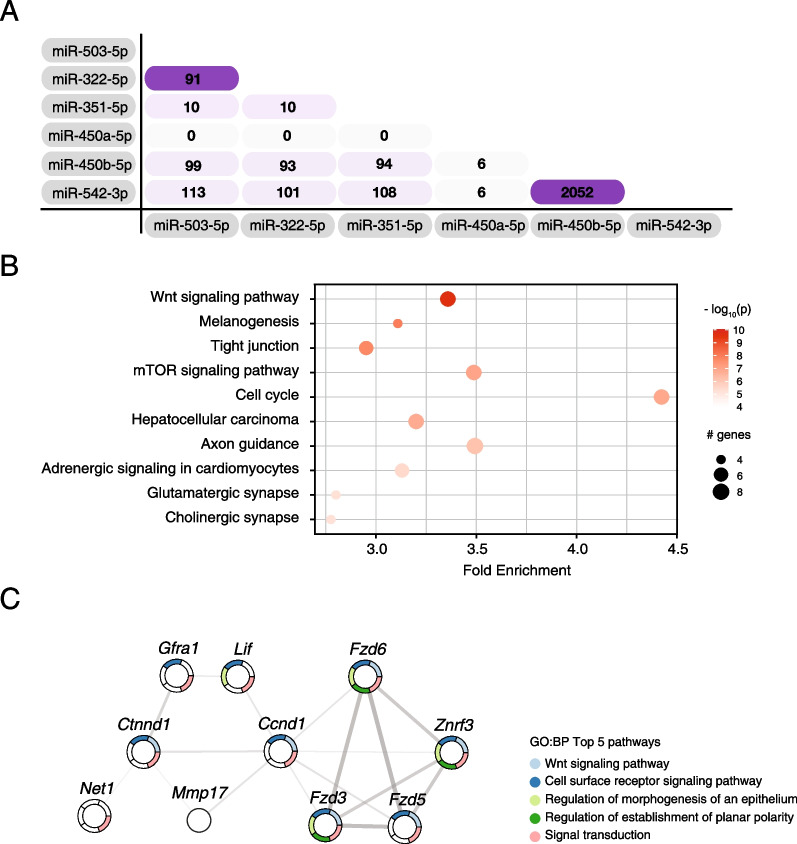
H19X-encoded miRNAs share target genes enriched in Wnt signaling pathways. **A** Analysis of shared target genes of H19X-encoded miRNAs using TargetScan. The numbers of overlapping target genes are presented in the table. **B** KEGG pathway analysis of genes that are downregulated by IL-4 and targeted by two or more miRNAs. **C** A functional module analysis for genes that are downregulated by IL-4 and targeted by two or more miRNAs. Among several cluster modules, a regulatory network of Wnt signaling–related genes is shown

To investigate the function of H19X-encoded miRNAs, we examined their target genes. Although both strands of miRNA duplexes were detected in small RNA sequencing results, only the major strands of mature miRNAs, that is, miR-322-5p, miR-503-5p, miR-351-5p, miR-542-3p, miR-450a-5p, and miR-450b-5p, were counted for the target analysis because the specific miRNA strand selected by Ago protein shows higher abundance and consequently tends to play a significant role in biological processes (Table 1) [26]. Through computational analysis, we found that six miRNAs shared a large set of mRNA targets (Fig. 4A). The overlapping targets of these miRNAs were 2355 genes, and 176 of these genes were targeted by more than three of the miRNAs. In particular, miR-322-5p and miR-503-5p, which both belong to the *miR-15* family, share 38.1% and 37.9% of their target genes, respectively. miR-450b-5p and miR-542-3p share 2052 target genes, accounting for 42.3% of miR-450b-5p target genes and 38.3% of miR-542-3p target genes. As a collaboration of miRNAs in the same cluster can show greater effects on target genes [37, 57], we investigated the physiological role of IL-4-upregulated miRNAs by focusing on shared target genes. Top-ranked KEGG pathways enriched in shared target genes indicated that these miRNAs may regulate Wnt and JAK-STAT signaling cooperatively (Additional file 1: Fig. S3). We then compared the shared target genes with the IL-4-downregulated genes. Among the 733 genes downregulated by IL-4, 381 genes were shared targets of six IL-4-upregulated miRNAs, which are enriched in the Wnt signaling pathway (Fig. 4B and Additional file 4: Table S3). In this gene set, Wnt signaling pathway genes constitute a functional module that forms the gene regulatory network (Fig. 4C).

### Wnt signaling–related genes, Ccnd1 and Fzd6, are downregulated by IL-4-upregulated miRNAs

**Fig. 5:**
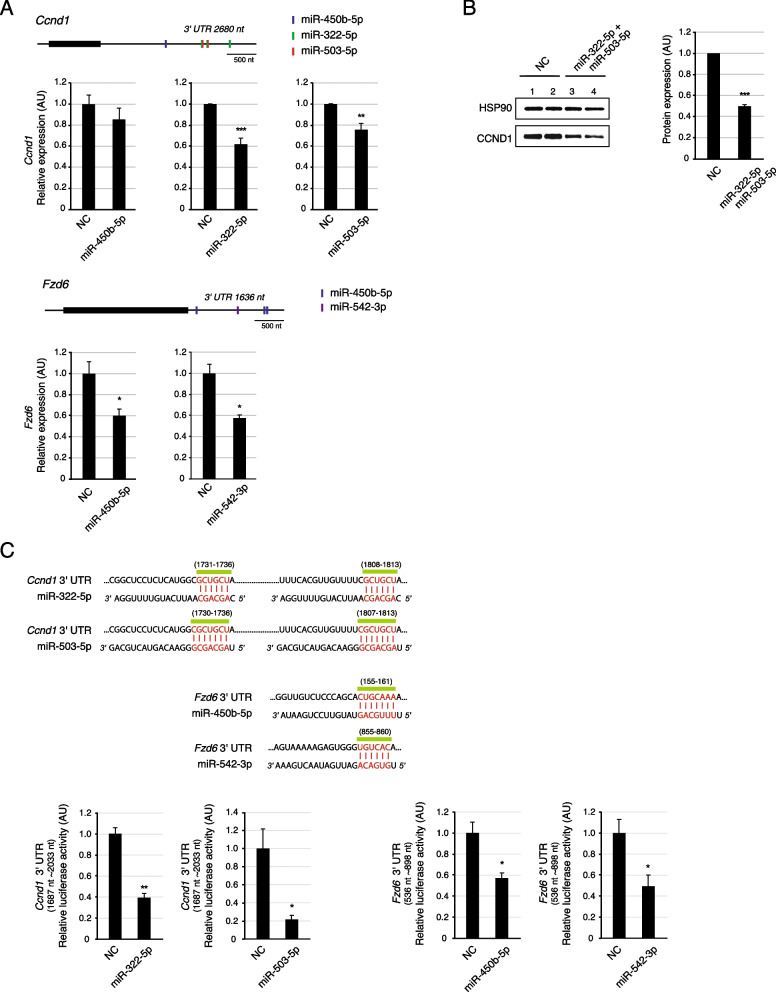
*Ccnd1* and *Fzd6* are repressed by H19X-encoded miRNAs. **A** The gene structures of *Ccnd1* and *Fzd6* are shown with binding sites for H19X-encoded miRNAs in the 3′ UTR. Ccnd1 mRNA expression was significantly repressed by mimic treatment of miR-322-5p or miR-503-5p, and not miR-450b-5p. Fzd6 mRNA levels were reduced by approximately 40% upon mimic treatment of miR-450b or miR-542-3p (n = 3). **B** The CCND1 protein levels decreased with miR-322-5p and miR-503-5p mimic treatment. The quantified signal intensities in the western blotting are presented as a bar graph (n = 2). **C** The positions and sequences of miRNA binding sites in the 3′ UTR of Ccnd1 and Fzd6 are presented (upper panel). The seed region of miRNA is highlighted in red and is base paired with the matched sequence of each target mRNA. The binding activity of miRNAs to the predicted sites in 3′ UTR was measured using a dual-luciferase reporter assay (n = 3). The luciferase activity was reduced by transfection of the corresponding miRNA when the reporter contained the 3′ UTR fragment of *Ccnd1* or *Fzd6. *Data are presented as mean ± SEM. ***p < 0.005, **p < 0.01, *p < 0.05

Next, we validated whether Wnt signaling–related genes were repressed by IL-4-upregulated miRNAs. Among the 10 genes of the cluster module (Fig. 4C), the expression levels of *Ccnd1* and *Fzd6* consistently decreased with the transfection of APs with the relevant miRNA (Fig. 5a and b). *Ccnd1*, which contains three binding sites for miR-322-5p, two binding sites for miR-503-5p, and one binding site for miR-450b in the 3′ UTR, was downregulated at both the mRNA and protein levels by miR-322-5p and miR-503-5p transfection (Fig. 5A and B). The mRNA levels of the *Fzd6* gene with multiple binding sites for miR-450b-5p and miR-542-3p were reduced by the relevant miRNAs (Fig. 5A). Direct targeting of miRNAs to the predicted binding sites was examined using luciferase reporter assays (Fig. 5C). The 3′UTR 1687–2033 nt region of *Ccnd1* was shown to induce the repression of luciferase activity upon miR-322-5p or miR-503-5p transfection when fused to the luciferase open reading frame. The 3′UTR 101–322 nt or 536–898 nt regions of *Fzd6* also decreased luciferase activity with miR-450b-5p or miR-542-3p transfection. These results indicate that IL-4-upregulated miRNAs directly regulate several Wnt signaling–related genes in APs.

### miRNA/Wnt regulatory feedback circuit controls the proliferation and commitment of APs

**Fig. 6:**
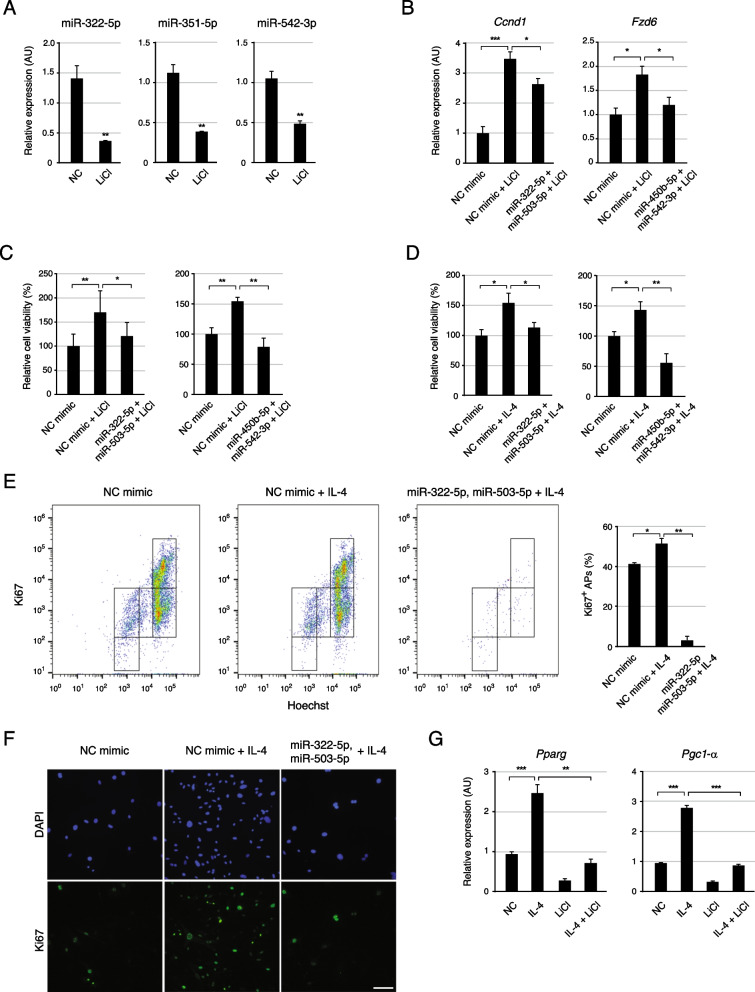
The negative feedback loop of miRNA/Wnt affects the proliferation and beige commitment in IL4-treated Aps. **A** LiCl treatment reduced the abundance of mature miRNAs to less than half that observed in control APs. miR-322-5p, miR-351-5p, and miR-542-3p were examined by qPCR as representatives of H19X-encoded miRNAs (n = 3). **B** The mRNA expression levels of Wnt signaling–related genes were measured by qPCR (n = 3). LiCl treatment increased the mRNA levels of *Ccnd1* and *Fzd6*. The transfection of miRNA mimics together with LiCl treatment alleviated the effect of the Wnt activator, LiCl. **C**, **D** The proliferation of APs was analyzed by MTT assay (n = 3). **C** LiCl treatment enhanced the cell proliferation, and transfection of miR-322-5p/miR-503-5p and miR-450b-5p/miR-542-3p mimics lowered the LiCl-induced proliferation. **D** IL-4-induced cell proliferation was suppressed by the transfection of miR-322-5p/miR-503-5p and miR-450b-5p/miR-542-3p mimics. **E**, **F** The proliferation of APs was analyzed by flow cytometry (**E**) and immunocytochemistry (**F**). The percentage of Ki67-positive cells increased after six days of incubation in IL-4 containing medium but decreased upon additional transfection of miR-322-5p/miR-503-5p mimics. Scale: 100 µm **G** The mRNA expression levels of beige adipogenic markers were measured by qPCR (n = 3). IL-4-induced upregulation of *Pparg* and *Pgc1-α* expressions was compromised by LiCl treatment. Data are presented as mean ± SEM. ***p < 0.005, **p < 0.01, *p < 0.05.

A previous study has found that the levels of miR-322-5p, miR-322-3p, miR-503-5p, miR-351-5p, and miR-542-5p decreased in the 3T3-L1 preadipocyte cell line upon treatment with the Wnt activator LiCl [58]. In addition, eight mature miRNAs were substantially downregulated, of which five were derived from the H19X genomic locus. Therefore, we evaluated the expression levels of miR-322-5p, miR-351-5p, and miR-542-3p after LiCl treatment in APs of scWAT and confirmed that their expression was downregulated by approximately 50% upon the activation of Wnt signaling (Fig. 6A). In addition, LiCl increased the expression of the Wnt signaling–related genes *Ccnd1* and *Fzd6*; this increase was attenuated by the addition of the relevant miRNAs (Fig. 6B). These results suggest that Wnt signaling–related genes and H19X-encoded miRNAs form a double-negative feedback loop in APs.

We then investigated whether the Wnt/miRNA regulatory circuit is involved in cellular expansion by MTT proliferation assay. LiCl increased the proliferation of APs by more than 1.5-fold compared to that observed in the non-treated control cells; however, miR-322-5p/miR-503-5p and miR-450b-5p/miR-542-3p suppressed the effect of LiCl (Fig. 6C). The IL-4-induced cellular expansion (~ 1.5 fold) was attenuated by miR-322-5p/miR-503-5p and miR-450b-5p/miR-542-3p (Fig. 6D). In addition, the regulatory effect of the miRNAs on IL-4-induced proliferation was also examined using APC staining coupled with Ki67, a nuclear marker for proliferating cells, followed by flow cytometry analysis. Indeed, IL-4 stimulation increased the AP cell number by 1.7 fold than that seen with the controls; however, this effect was abolished in the presence of the miR-322-5p and miR-503-5p mimics (Additional file 1: Fig. S4A). Similarly, immunofluorescence imaging revealed that Ki67-positive proliferating cells constituted 54% in IL-4–treated APs and 41.9% in control cells. However, in APs treated with IL-4 and miR-322-5p/miR-503-5p mimics, Ki67-positive cells constituted only 4.5% (Fig. 6E). The suppression of IL-4 induced proliferation by miR-322-5p and miR-503-5p mimics was also confirmed by immunocytochemistry (Fig. 6F). Next, we examined the effect of Wnt signaling on the beige commitment of bipotential APs. The IL-4-mediated commitment to beige adipocyte lineage was abolished by LiCl-induced Wnt signaling activation (Fig. 6G and Additional file 1: Fig.S4B). These results indicate that the Wnt/miRNA regulatory circuit controls the proliferation and commitment of APs under type 2 cytokine stimulation.

### H19X-encoded miRNAs affect the differentiation potential of APs

**Fig. 7:**
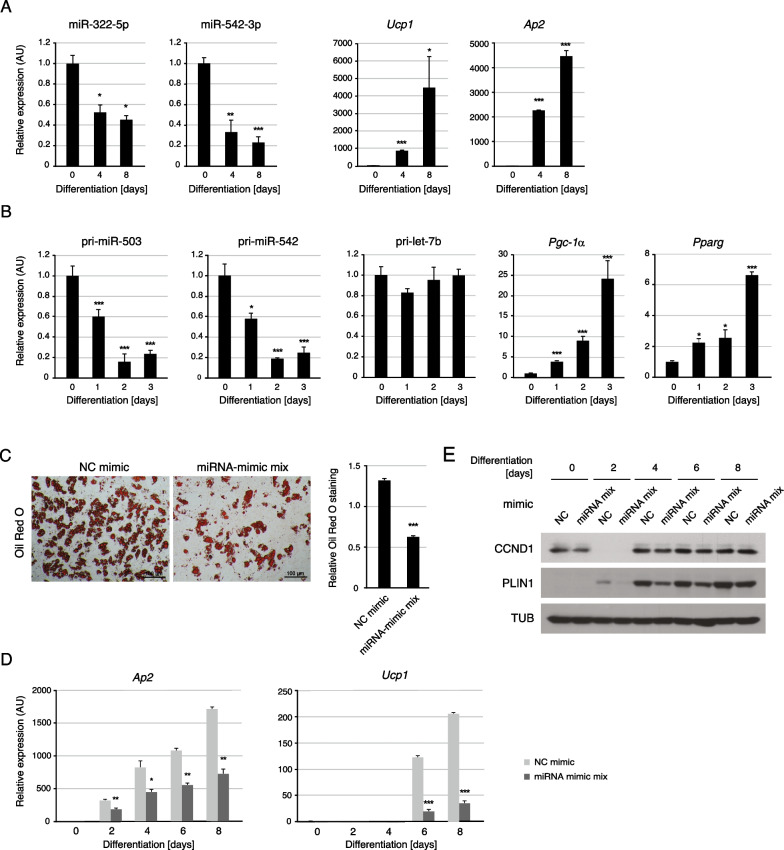
Dysregulation of H19X-encoded miRNAs in APs affects their differentiation into beige adipocytes. **A**, **B** Cells were cultured in a differentiation medium for beige fat lineage and harvested for analysis at several time points (n = 3). **A** Mature miRNAs, miR-322-5p and miR-542-3p, were downregulated in differentiated beige adipocytes. **B** The primary transcripts of *miR-503* and *miR-542* were downregulated immediately upon the differentiation of adipocytes. The *let-7b* primary transcript was used as a control. **C**-**E** APs were transiently transfected with miRNA mimic mix (miR-322-5p/miR-503-5p/miR-450b-5p/miR-542-3p) for 24 h and stimulated with an adipogenic cocktail for the differentiation. Cells were harvested for analysis at several time points after the induction of differentiation (day 0, 2, 4, 6, and 8) (**C**) The representative images of Oil Red O staining in differentiated beige adipocytes at day 8 (left panel). The quantified intensities of Oil Red O staining are shown (right panel). The pretreatment of miRNA mimic mix reduced the number of differentiated adipocytes. Scale bar, 100 µm (**D**) The mRNA levels of *Ucp1* and *Ap2* were analyzed during beige adipocyte differentiation (n = 2). The pretreatment of miRNA mimic mix (miR-322-5p/miR-503-5p/miR-450b-5p/miR-542-3p) attenuated the expression of *Ucp1* and *Ap2*. **E** The protein levels of CCND1 and PLIN1 were examined by western blotting during beige adipocyte differentiation. miRNA mimic mix downregulated CCND1 protein levels and delayed the expression of PLIN1 protein. Data are presented as mean ± SEM. ***p < 0.005, **p < 0.01, *p < 0.05

H19X-encoded miRNAs were more abundant in APs than in the adipocyte differentiation state (Fig. 7A). The primary transcripts of *miR-503* and *miR-542* were rapidly downregulated after the induction of differentiation (Fig. 7B). In another study, the cluster of *miR-322/-503* was reported to be transcriptionally repressed during adipogenesis [59]. During postnatal mouse development, pri-miR-503 and pri-miR-542 transcripts showed higher expression levels one week after birth when the IL-4 level was elevated in scWAT and decreased with the accumulation of mature adipocytes, which is indicated by increasing Ap2 levels (Additional file 1: Fig. S5). The expression patterns of H19X-encoded miRNAs indicate that they primarily play a role in the undifferentiated state of APs.

Next, we addressed whether the function of H19X-encoded miRNAs in undifferentiated APs affects the following progress of adipocyte differentiation. When APs were pretreated with miRNA mimics 2 days before induction of differentiation, in which CCND1 protein levels were decreased at the start point of differentiation (differentiation day 0), adipocyte differentiation was compromised (Fig. 7C–E). At the molecular level, *Ap2* mRNA, *Ucp1* mRNA and PLIN1 protein levels were lower in the miRNA mimic-treated samples than in the control group at all-time points of differentiation (Fig. 7D and E). Note that CCND1 protein levels fluctuated during differentiation in the control group, which may reflect the dynamic regulation for balancing proliferative cells and differentiating cells in adipogenesis (Fig. 7E) [60]. Overall, the overexpression of H19X-encoded miRNAs in the transition from adipocyte precursors to differentiated adipocytes altered both proliferation and adipogenic markers and attenuated adipogenesis.

## Discussion

The regulation of adipogenesis is critical for the formation and maintenance of adipose tissue [61]. The number, type, and maturation of adipocytes determine the function of adipose tissue, which affects systemic metabolism [62]. Here, we identified a novel mechanism controlling the proliferation and commitment of APs during the initial step of adipogenesis (Additional file 1: Fig. S6). Different types of progenitor cells found in diverse tissues show active proliferation, facilitating cellular expansion [63]. Upon stimuli, including developmental cues, these cells undergo growth arrest, cell specification, and further differentiation [64]. However, the molecular mechanisms underlying the transition from proliferation to differentiation are not completely understood. Recently, the miR-15 family of miRNAs was reported to play an important role in regulating this transition in precursor B lymphocytes [65]. In early B cell development, the biphasic sequence of proliferation and cell fate decision is controlled by the miR-15 family through a regulatory circuit involving IL-7R and pre-BCR signaling [66]. Our study also found that the *miR-322/-503* cluster of the miR-15 family contributes to the transition of APs from proliferation to differentiation under IL-4 signaling. Together with the previous study, our findings highlight the regulatory role of the miR-15 family during adipocyte development and suggest a potential role for the miR-15 family in cellular transition across diverse biological contexts.

H19X-encoded miRNAs, especially *miR-322/-503* are known to regulate fundamental biological processes including cell cycle, apoptosis, and stress response, and play a role in the cell fate specification and differentiation of cardiomyocytes and skeletal muscle and monocytes [67–71]. In this study, we showed that the overexpression of H19X-encoded miRNAs attenuates differentiation in APs, which is the opposite phenotype of *miR-322/-503* knockout mice [59]. Specifically, Rodríguez-Barrueco et al. observed fat mass expansion in *miR-322/-503* knockout mice and an increase in adipogenic progenitors and hypertrophy with a high-fat diet and reported the enhanced secretion by adipocytes of γ-synuclein, which is encoded by a target gene of *miR-322/-503*, mediates altered adipocyte differentiation and adipose tissue enlargement in knockout mice [57]. However, given the sharp downregulation of miR-322/-503 expression during differentiation, the role of miR-322/-503 may be critical in APs but less significant in mature adipocytes.

In our study, H19X-encoded miRNAs in mouse scWAT were expressed at high levels one week after birth. The levels of miRNA primary transcripts decreased as differentiated adipocyte markers increased over several weeks of postnatal development. Interestingly, IL-4 expression also reached the highest level in the postnatal one-week scWAT sample. In noninflammatory contexts, eosinophils that secrete IL-4 are found in several tissues, including the gastrointestinal tract, lungs, adipose tissue, thymus, uterus, and mammary glands [72]. High eosinophil activity is linked to developmental and morphogenetic events during postnatal development [73, 74]. Our data suggest that eosinophil-derived IL-4 stimulation and consequent miRNA/Wnt feedback regulation may be involved in the postnatal development of adipose tissue in vivo. In the future, it will be interesting to address whether IL-4 stimulates cellular expansion and beige commitment in APs and promotes the browning of adipocytes immediately after birth.

In the development and remodeling of adipose tissue, stepwise procedures of cellular transition, such as growth arrest, clonal expansion, and differentiation, have been widely studied, predominantly using in vitro systems [75]. Although in vitro studies cannot completely mimic in vivo adipogenesis, they have enabled us to understand the key regulators and signaling cascades involved in stem cell proliferation, adipocyte lineage commitment, and differentiation. Our study revealed that miRNA-mediated regulation of the Wnt signaling pathway is a mechanism by which type 2 immunity controls the cellular expansion and commitment of APs in vitro. Moreover, we suggested the possibility that this regulation is developmentally programmed to facilitate beige fat biogenesis. Advances in experimental tools, including single-cell sequencing, will provide in vivo evidence in future studies, which are expected to establish the Wnt/miRNA regulatory circuit in the immunomodulation of APs.

## Materials and methods

### Adipocyte progenitor (AP) isolation and culture

Subcutaneous WAT was digested in collagenase I buffer (2 mg/mL at 250 U/mg, Worthington) and 30 mg/mL bovine serum albumin in Hams F-10 medium at 37 °C for 20–30 min. The homogenates were filtered (40 mm) and APs were isolated using MACS (Miltenyi) through the negative selection of CD45 and CD31, followed by positive selection for SCA-1 as described previously (Lee et al., Cell, 2015). APs were cultured in DMEM F12 supplemented with 10% fetal bovine serum (Gibco). Cells were plated at 1 × 10^5^ cells/well in 12-well plates for IL-4 or LiCl treatment and oligo transfection. RNA oligonucleotides were transfected at 40 nM into each cell line using Lipofectamine RNAiMAX (Invitrogen) according to the manufacturer’s protocol. LiCl treatment and oligonucleotide transfection samples were analyzed after two-day incubation. Unless otherwise indicated, APs were incubated in IL-4-containing media for four days and subsequently processed for analysis.

### Quantitative RT-PCR

Total RNA was extracted from cells using TRIzol reagent and treated with DNase I. For mRNA, cDNA was synthesized with 0.1–0.5 μg of total RNA using a reverse transcription kit, the PrimeScript RT-PCR kit (Takara), or ReverTra Ace qPCR RT Master Mix (Toyobo). mRNA levels were analyzed by quantitative PCR using the synthesized cDNA and normalized to the levels of 36B4. qPCR was performed using SYBR Green PCR Master Mix (Applied Biosystems) or SYBR Green real-time PCR Master Mix (Toyobo). Mature miRNA levels were determined using TaqMan MicroRNA Assays (Applied Biosystems) and normalized to the levels of U6 snRNA. The comparative cycle threshold (Ct) method was used to determine the relative miRNA and mRNA levels.

### Western blotting

Cells were lysed in a modified RIPA buffer (420 mM NaCl, 1% NP-40, 0.1% SDS, 0.5% sodium deoxycholate, 50 mM Tris pH 7.5, and protease inhibitor cocktail). Each protein sample (35–50 μg) was separated on 4–12% SDS-PAGE gels and transferred to a nitrocellulose membrane (GE Healthcare). The membranes were blocked in PBS-T (phosphate-buffered saline, 0.1% Tween 20) containing 3% bovine serum albumin (BSA) and subsequently probed with primary antibodies and HRP-conjugated secondary antibodies. Protein levels were detected with the SuperSignal West Pico Chemiluminescent Substrate (Thermo Scientific). Anti-CCND1(#2922, CST), anti-UCP1 (ab10983, Abcam), anti-TUB (ab52866, Abcam), anti-PLIN1 (P1998, Sigma), and anti-HSP90 (#4874, CST) antibodies were used as the primary antibodies.

### Luciferase assay

Firefly luciferase reporter plasmids were generated for luciferase assays. The mouse *Ccnd1* 3′ UTR (1687–2033 nt) and *Fzd6* 3′ UTR (101–322 nt, 536–898 nt) regions were amplified by PCR from genomic DNA of adipocyte precursor cells and cloned into the pGL3 3′UTR vector (Kim et al., NSMB, 2009). Firefly luciferase reporter plasmids were transiently transfected with miRNA mimics in HEK 293 T cells. The Renilla luciferase plasmid was co-transfected to normalize the transfection efficiency. Two days after transfection, the cells were harvested, and a dual-luciferase assay (Promega) was performed according to the manufacturer’s protocol. Firefly luciferase activity levels were normalized to the Renilla luciferase activity. The data are presented as relative luciferase activity (luciferase 3′UTR reporter activity/luciferase control reporter activity). All assays were performed at least three times.

### MTT cell proliferation assay

The cell proliferation rate was determined using MTT (3-(4,5-Dimethylthiazol-2-yl)-2,5-diphenyltetrazolium bromide) assay. Adipocyte precursor cells were cultured in 96-well plates for 24 h, and the medium was replaced with an MTT reagent (Roche). After 4 h, dimethyl sulfoxide (DMSO) was added to each well and incubated for 30 min. The absorbance was measured at 570 nm using a microplate reader (Epoch Biotek Microplate Spectrophotometer).

### Flow cytometry analysis

Undifferentiated APs were cultivated on cell culture dishes with Dulbecco’s Modified Eagle Medium F12 supplemented with 10% fetal bovine serum (Gibco) containing IL-4 alone or in combination with transfection of miR-322-5p and miR-503-5p for 6 days to cell expansion. Cells were washed with PBS and then dissociated to a single-cell suspension by trypsin treatment for 3–5 min. The reaction was stopped with culture medium. After centrifugation, the cells were washed in PBS and fixed with 4% paraformaldehyde for 15 min at room temperature. Cells were permeabilized with ice-cold 90% methanol for 30 min on ice, and then immunostained using Ki67 monoclonal antibody (50–5698-82, eBioscience) and Hoechst 33,342. Flow cytometric quantitations of Ki67^+^/ Hoechst 33,342^+^ cell populations were performed by analysis of duplicate.

### Immunofluorescence staining

APs were isolated from scWAT and cultivated with cell culture medium containing IL-4 alone or in combination with transfection of miR-322-5p and miR-503-5p for 6 days to cell expansion. Cells were fixed with 4% paraformaldehyde for 10 min, permeabilized with 0.1% Triton™ X-100 for 10 min, washed in PBS three times, and then blocked with 2% BSA for 1 h at room temperature. The cells were labeled with Ki67 monoclonal antibody (SP6) (MA5-14,520, Thermo Fisher Scientific) at 1:250 dilution in 0.1% BSA, incubated at 4 °C overnight and then labeled with donkey anti-rabbit IgG (H + L) highly cross-adsorbed secondary antibody, Alexa Fluor Plus 488 (A11034, Thermo Fisher Scientific) at a dilution of 1:2000 for 60 min at room temperature (green). Nuclei (blue) were stained using Antifade Mounting Medium and DAPI (H-1500, Vector Laboratories).

### mRNA library preparation and sequencing

RNA was extracted from APs using TRIzol (Invitrogen) and mRNAs were purified using poly T oligo-attached magnetic beads. The quality and quantity of the RNA samples were assessed using an Agilent 2100 Bioanalyzer (Agilent RNA 6000 Nano Kit). RNA-seq libraries were constructed by BGI Genomics Co., Ltd., and sequenced on the BGISEQ-500 platform. Low-quality raw sequence reads were filtered by SOAPnuke software to obtain clean reads. Filtered reads were mapped to the mouse reference genome (*Mus musculus*, UCSC mm10) using Hierarchical Indexing for Spliced Alignment of Transcripts.

### Small RNA library preparation and sequencing

Two biological replicates of control and IL-4–treated APs were prepared. Small RNA-seq libraries were generated with 3 µg total RNA using the TruSeq Small RNA Library Preparation Kit (Illumina), and cDNA fragments were sequenced on the Illumina HiSeq 2500 platform. Raw sequencing reads were trimmed by removing the adapter sequence and were filtered using a Phred quality score. Unique clustered reads were sequentially aligned to the mouse reference genome, miRBase v21, and the non-coding RNA database, Rfam9.1. Read counts for each miRNA were extracted to determine the abundance of each miRNA from the miRDeep2 Quantifier module.

### Differential expression analyses

To analyze differentially expressed (DE) miRNAs, log2 transformation of Count + 1 and quantile normalization was performed. Statistical analysis was performed using fold change and nbinomWaldTest with DESeq2 per comparison pair. The significant DE results were selected on the conditions of FC > 1.5 and nbinomWaldTest p < 0.05. For differentially expressed gene (DEG) analysis of RNA-seq data, Bowtie2 and RSEM tools were used to calculate the gene expression levels for each sample. DEGs were detected with baseMean (average of the normalized counts divided by size factors) generated from the DEseq2 algorithm (log2FoldChange (Sample2/Sample1) > 1 and adjusted p-value < 0.05). Volcano plots for mRNA and small RNA sequencing data were visualized using the R package, EnhancedVolcano v1.12.

### miRNA target prediction/KEGG pathway enrichment/miRNA-mRNA regulatory network/transcription factor (TF) enrichment analyses

miRNA target prediction analysis was performed using TargetScan-Mouse v7.2 [76]. The shared target genes of two different miRNAs were plotted using FunRich v3.1.3 software [77]. KEGG (Annotation and Kyoto Encyclopedia of Genes and Genomes) pathway enrichment analysis was conducted with DEGs from RNA-seq data using the R package, pathfindR. p-value < 0.05 was considered the cut-off criterion. For regulatory network analysis, the STRING (Search Tool for the Retrieval of Interacting Genes/Proteins) database (stringApp v1.7.1) was used with a confidence score cut-off of > 0.2 [78]. The gene regulatory network was visualized with Cytoscape v3.9.0-BETA1 [79]. The Cytoscape plugin MCODE v2.0, was used to calculate key hub genes in the constructed gene regulatory network [80]. Significant functional modules were identified using the Cytoscape plugin cytoHubba v0.1 [81]. TFs were predicted for the promoter region of H19X-encoded miRNAs (1.5 kb upstream of the TSS and ending 0.5 kb downstream of TSS) using microTSS [56]. Next, we performed TF enrichment analysis using the R package, PWMEnrich [82].

### Statistical analysis

All experimental data are presented as mean ± standard error of the mean (SEM). Student’s t-tests were used to analyze the significance of differences using the GraphPad Prism 8 software (GraphPad). Statistical significance was set at p < 0.05 (*** *p* < 0.005, ** p < 0.01, * p < 0.05).

## Supplementary Information

**Additional file 1. Figures s1-s6**

**Additional file 2. Table s1**

**Additional file. 3. Table s2**

**Additional file 4. Table s3**

## Data Availability

RNA sequencing data generated in this study have been deposited in the Gene Expression Omnibus (GEO) with the accession number GSE216077.
