# Supplementary material for: H19X-encoded microRNAs induced by IL-4 in adipocyte precursors regulate proliferation to facilitate differentiation
Source: Biol Direct. 2023 Jun 15;18:32. doi: 10.1186/s13062-023-00388-4 (PMC10273709; doi:10.1186/s13062-023-00388-4)
Supplement: Supplementary file 1 — Additional file 1. Figures s1-s6 [file 13062_2023_388_MOESM1_ESM.pdf]

Supplementary Figure 1

A

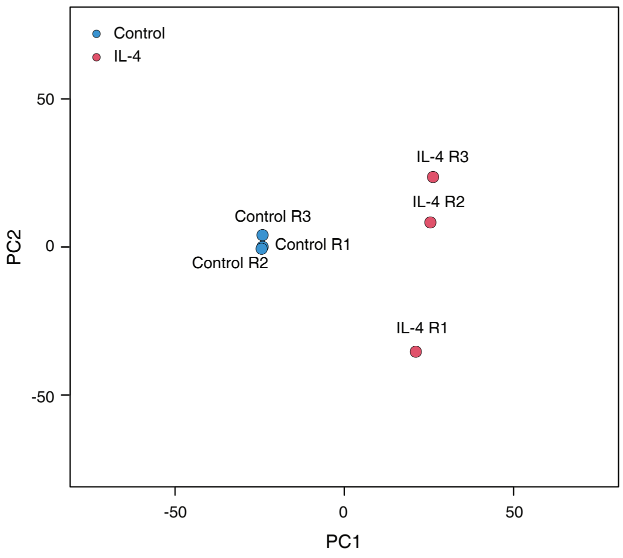

B

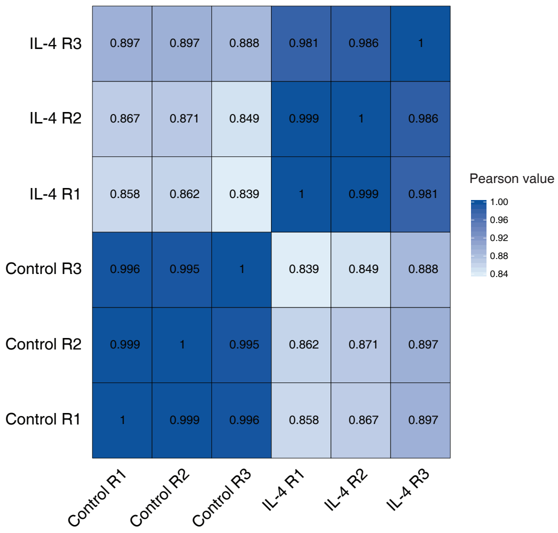

Fig. S1. mRNA sequencing data analysis

(A) Principal component analysis (PCA) of three replicates for control and IL-4–stimulated adipocyte precursors. (B) Correlation matrix of all the samples.

Supplementary Figure 2

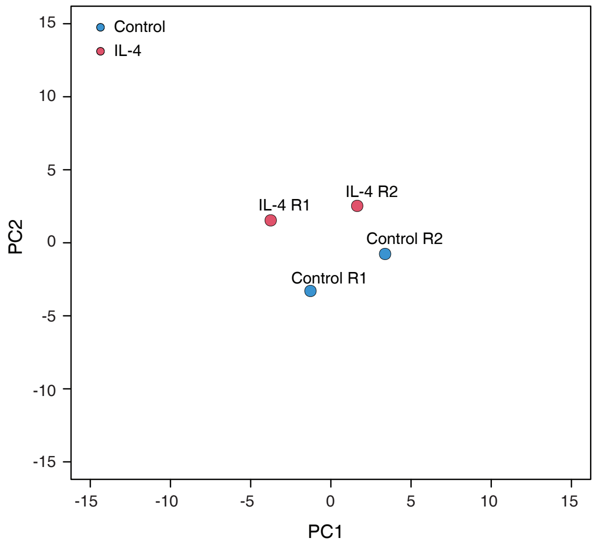

Fig. S2. small RNA sequencing data analysis

Principal component analysis (PCA) of two replicates for control and IL-4–stimulated adipocyte precursors.

Supplementary Figure 3

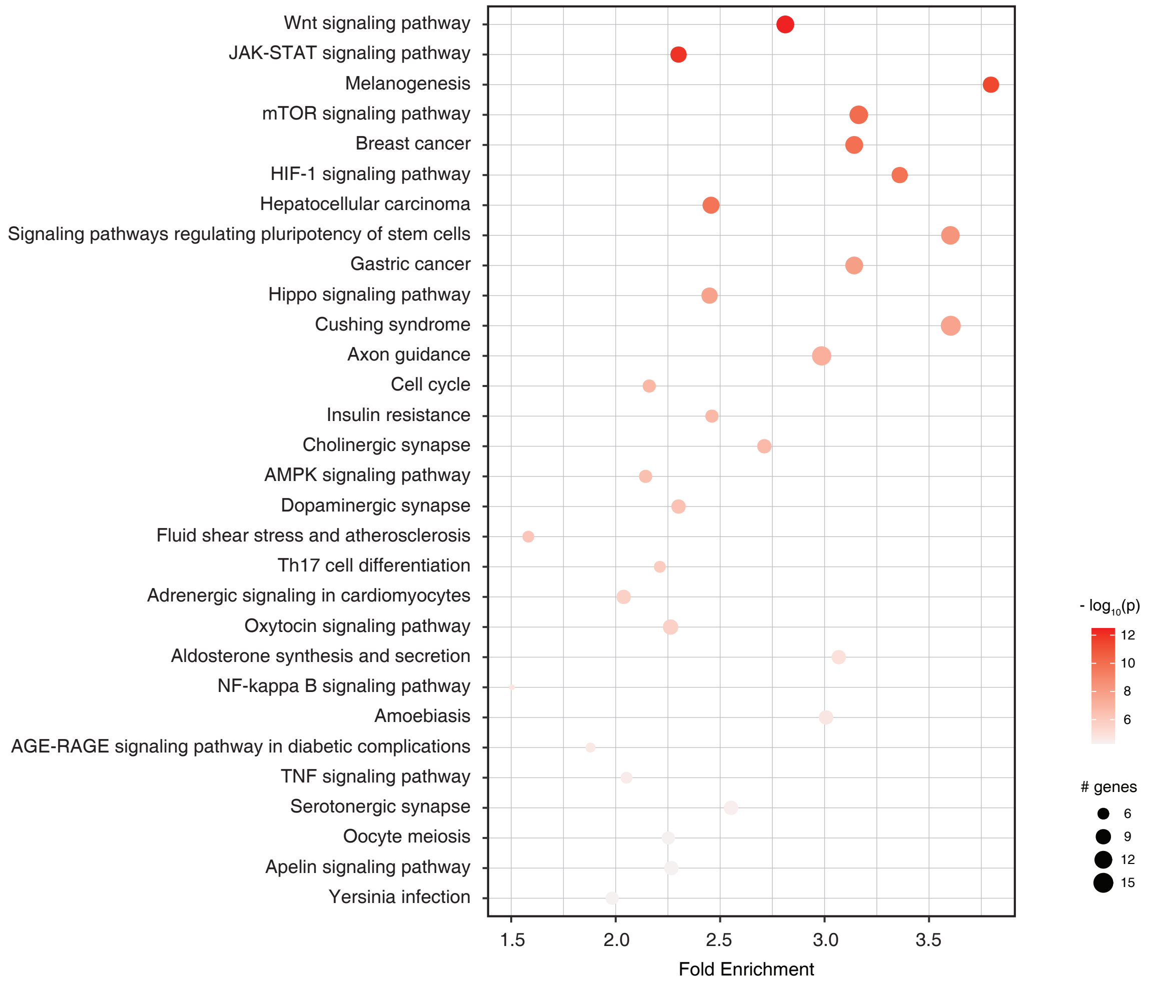

Fig. S3. KEGG pathway analysis in shared target genes of H19X-encoded miRNAs

Supplementary Figure 4

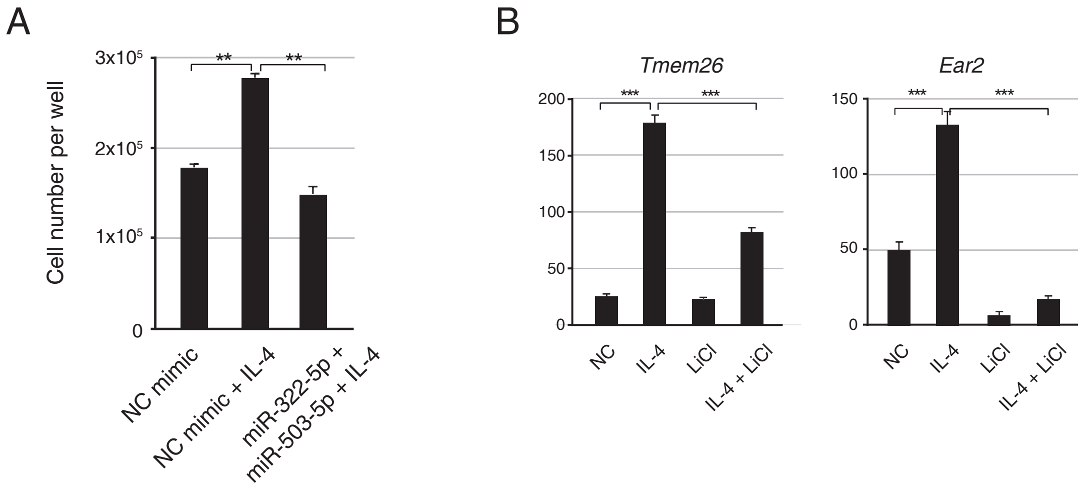

Fig. S4. H19X-encoded miRNAs and Wnt activator, LiCl, affect IL-4 induced proliferation and beige commitment in adipocyte precursors (APs)

(A) Total cell number analysis of adipocyte precursors. Adipocyte precursors were counted for each condition (control, IL-4, IL-4+ miR-322-5p/miR-503-5p) (n=2). (B) The mRNA expression levels of beige adipogenic markers were measured by qPCR (n=3). IL-4-induced upregulation of *Tmem26* and *Ear2* expressions was compromised by LiCl treatment. Data are presented as mean ± SEM. *** $p < 0.005$, ** $p < 0.01$, * $p < 0.05$.

Supplementary Figure 5

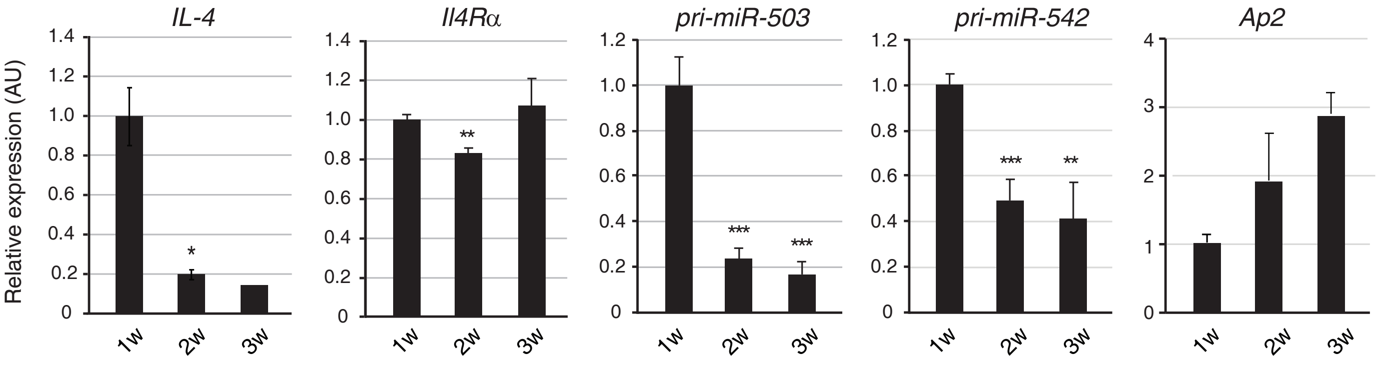

Fig. S5. H19X-encoded miRNAs are highly expressed at birth and gradually downregulated during postnatal development in mice

mRNA expression levels of *IL-4*, *IL-4Rα*, and *Ap2* were measured via qPCR in scWAT samples of 1, 2, and 3 week postnatal mice (n=3). IL-4 mRNA level was below the detection level in two samples of 3-week-old mice and measured in only one sample (n=1). Expression levels of the primary transcripts of H19X-encoded miRNAs decrease, whereas that of the adipogenic marker *Ap2* increases during the course of postnatal development.

Supplementary Figure 6

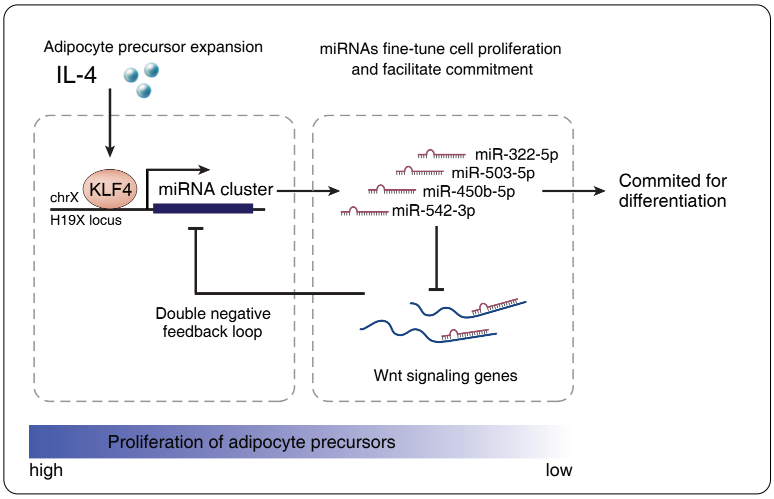

Fig. S6. A proposed model of H19X-encoded miRNAs-mediated regulation of adipocyte precursors under IL-4 stimulation

H19X-encoded miRNAs are upregulated upon IL-4 stimulation in adipocyte precursors through the transcription factor KLF4. H19X-encoded miRNAs that are downregulated by Wnt signaling activation suppress the expression of Wnt signaling pathway genes. The miRNA/Wnt double-negative feedback loop lowers the proliferation of adipocyte precursors induced by IL-4 stimulation and contributes to beige adipocyte commitment.
